# Evaluation of Efficacy and Safety of Dan'e-Fukang Soft Extract in the Treatment of Endometriosis: A Meta-Analysis of 39 Randomized Controlled Trials Enrolling 5442 Patients

**DOI:** 10.1155/2017/9767391

**Published:** 2017-02-27

**Authors:** Yantao Li, Te Li, Shilin Song

**Affiliations:** ^1^Department of Prevention, Nankai Hospital, Tianjin Academy of Integrative Medicine, Tianjin 300100, China; ^2^Department of Chinese Medicine, Tianjin Hearing Impairment Specialist Hospital, Tianjin 300150, China; ^3^Laboratory of Anatomy, School of Integrative Medicine, Tianjin University of Traditional Chinese Medicine, Tianjin 300193, China

## Abstract

*Objective*. To systematically evaluate the efficacy and safety of Dan'e-fukang soft extract in endometriosis treatment.* Method*. PubMed, CNKI, Wanfang Database, VIP, SinoMed, and Cochrane Library were searched. Randomized controlled trials (RCTs) comparing the efficacy of Dan'e-fukang soft extract and conventional western medicines for endometriosis treatment were included. The data were extracted independently by two people and analyzed using RevMan 5.2.0 software. The relative risk (RR) and mean difference (MD) with 95% confidence intervals were considered as effective outcome indicators.* Results*. Thirty-nine papers including 5442 patients with endometriosis were included in this study. A meta-analysis revealed that Dan'e-fukang soft extract was more efficient than gestrinone in the treatment of endometriosis (RR = 1.08, 95% CI = 1.03 to 1.15, *I*^2^ = 71%, REM, 18 trials) and its efficacy was comparable to that of danazol and mifepristone. Dan'e-fukang soft extract was also as effective as gestrinone and mifepristone in terms of relapse rate and relieving dysmenorrhea. The incidence of adverse reactions was lower than that of conventional western medicines.* Conclusions*. The results of this study showed that Dan'e-fukang soft extract offers certain advantages in endometriosis treatment, but rigorously designed, strictly implemented RCTs are needed to further validate its efficacy.

## 1. Introduction

Endometriosis (EMs) is an estrogen-dependent gynecological disease in which endometrium-like tissues grows in abnormal sites other than the endometrium, and it can lead to infertility and dysmenorrhea [1]. The global prevalence of endometriosis in women of childbearing age is about 5%–15% [2]. Ectopic lesions at various positions, most commonly in the ovary, and extensive adhesion of pelvic tissues can occur. Laparoscopic surgery is the first choice in the treatment of endometriosis. However, endometriosis treatment without total excision may cause hyperplasia, which leads to recurrence, and the recurrence rate is up to 40% [3]. The drugs currently used in western medicine include danazol, gestrinone, and mifepristone. However, these drugs have serious side effects, and therefore, there is an urgent need to find new, safe, and effective drugs to treat this disease.

According to traditional Chinese medicine (TCM), the basic pathogenesis of endometriosis is blood stasis. Therefore, the primary therapy is promoting blood circulation to remove blood stasis. Dan'e-fukang soft extract is the first pure TCM drug that received national drug approval number for the treatment of endometriosis. It consists of* Salvia*,* Rhizoma Curcumae*,* Rhizoma Sparganii*, bupleurum root, angelica, liquorice,* Rhizoma Corydalis*,* Radix Paeoniae Rubra*, and* Rhizoma Cyperi*. Most of the constituents in the prescription disseminate into the hepatic circulation and is involved in coordinating qi and blood, promoting blood circulation to remove blood stasis, and relieving pain. Animal experiments have shown that Dan'e-fukang soft extract helps in modulating immune functions by enhancing cellular immunity and reducing humoral immunity [4]. Currently, Dan'e-fukang soft extract is widely used in clinical practice in China, but it lacks effective evidence-based support. This study will systemically evaluate the clinical efficacy and safety of Dan'e-fukang soft extract in the treatment of endometriosis to provide a reference for clinical management.

## 2. Methods

### 2.1. Inclusion Criteria

*Inclusion Criteria*. (1) Type of study is as follows: randomized controlled trial (RCT), either blinding or using placebo, and languages limited to Chinese and English. (2) Study candidates are patients who received a diagnosis of endometriosis; endometriosis sites and patients receiving surgeries are not limited. (3) Interventions are as follows: Dan'e-fukang soft extract treatment alone in the test group; medication doses and treatment are not limited; the control group may include patients without treatment or patients receiving placebo or conventional western medicines. The courses of treatment, methods, and dosage are not limited. (4) Outcome measures are as follows: efficacy rate, recurrence rate, remission rate of dysmenorrhea, CA125, and safety.

### 2.2. Search Strategies

PubMed, Cochrane Library, VIP, CNKI, Wanfang Database were searched by computer. The search period ranged from their date of foundation to July 1, 2016. The search terms included Dan'e-fukang soft extract (dan e fu kang jian gao), endometriosis, and random. The references to included literatures were also searched.

### 2.3. Literature Screening

The titles and abstracts of the searched papers were studied by two independent investigators. Trials not obviously meeting the inclusion criteria were excluded. For trials that possibly met the inclusion criteria, the entire papers were further studied to determine if they actually met the inclusion criteria, and they were crosschecked. Disagreements on whether certain trials should be included were resolved through discussions.

### 2.4. Methodological Quality Assessments for Included Studies

Methods recommended by the Cochrane Collaboration were adopted in the methodological quality assessment for RCT, which included six risks of bias assessments: random allocation sequence generation, allocation concealment, blinding, data integrity, selective outcome reporting, and other biases [5]. Entries were considered to have a low risk of bias (low) if they met the criteria and a high risk of bias (high) if they did not meet the criteria. In addition, entries were considered unclear when the paper did not provide enough information for judgment. Methodological quality assessment of clinical trials was performed by two reviewers independently and all disagreements were resolved through discussions.

### 2.5. Data Extraction and Analysis

The two researchers employed unified data extraction forms and independently extracted data, which included general characteristics of the patient, diagnostic criteria, interventions, follow-up, and efficacy evaluation indicators.

RevMan 5.2.0 software from the Cochrane Collaboration was used for meta-analysis. Relative risk (RR) was used for count data, mean difference (MD) was used for quantity data, and 95% confidence interval (CI) was used to ascertain the range of the results. The chi-square test was used for assessing the heterogeneity of all clinical trials. For the test of heterogeneity (*I*^2^ > 50%, *p* < 0.1), random effects model (REM) was used to analyze the expression effect, whereas a fixed effects model (FEM) was used to merge data.

## 3. Results

### 3.1. Process for Including Literatures

**Figure 1:**
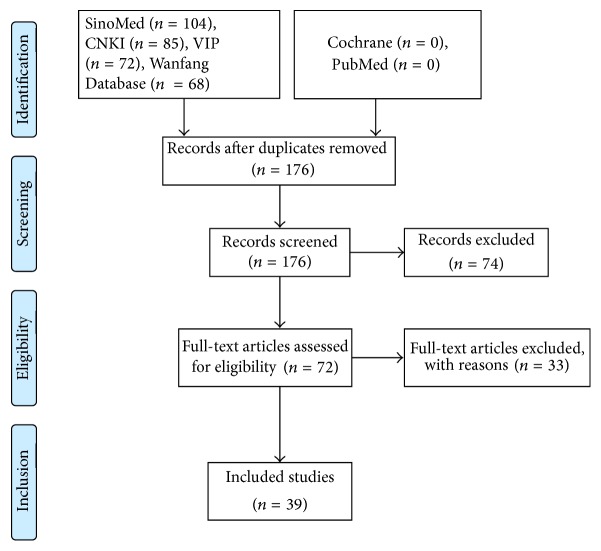
PRISMA 2009 flow diagram.

Thirty-nine papers were included in this study [1–39]. The literature searching process is shown in Figure 1.

### 3.2. Characteristics of the Included Studies

5442 endometriosis patients mentioned in the 39 papers were included in this study, with an average sample size of 140 cases. All papers were published Chinese literatures in mainland China. Dan'e-fukang soft extract alone was used in the 39 papers. The control groups were danazol, gestrinone, mifepristone, and marvelon. Laparoscopic postoperative medication was used in 9 papers [8, 13, 17, 25, 26, 31, 39–41]. The features of the included literatures are shown in Table 1.

### 3.3. The Methodological Quality of Included Studies

**Figure 2:**
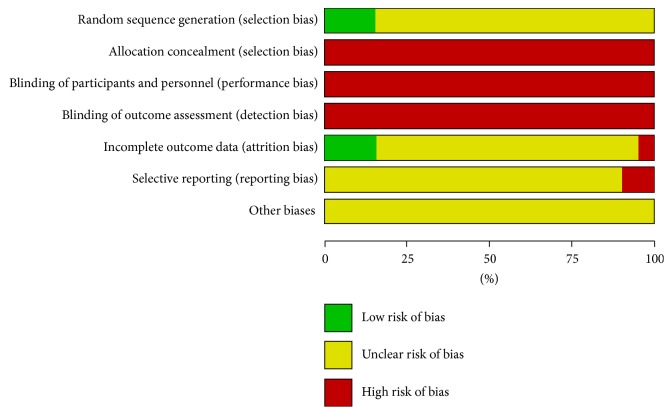
Risk of bias graph.

**Figure 3:**
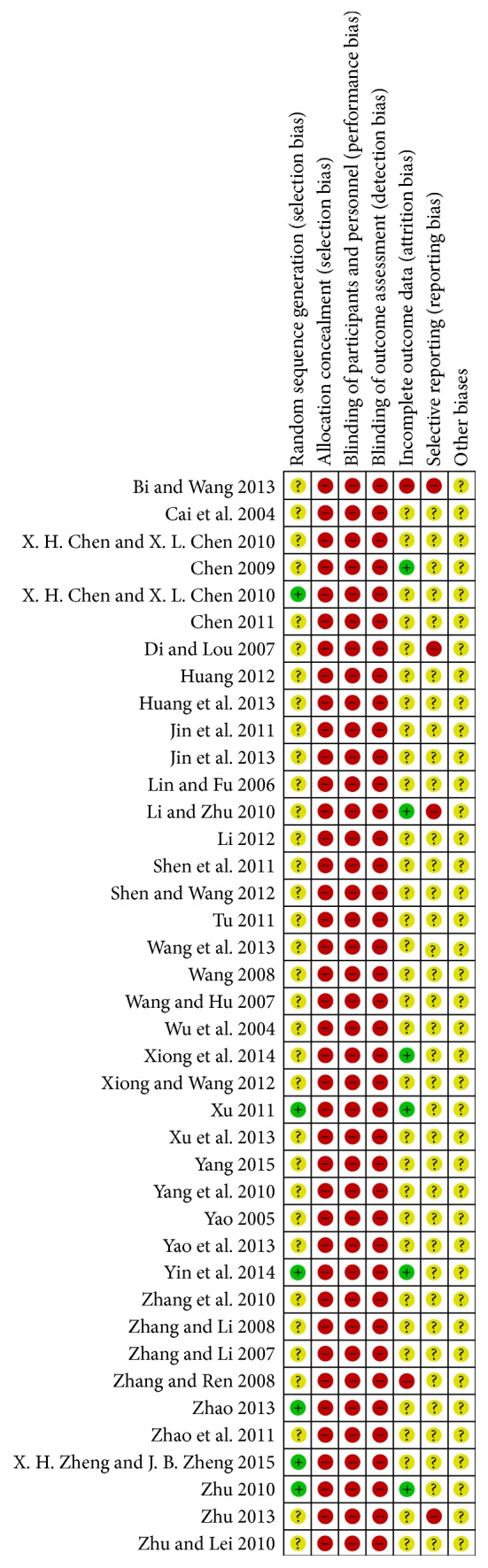
Risk of bias summary.

The studies were biased with high risks and the quality was low in all included papers. Wherein six papers used a random number table to randomize the groups [9, 28, 34, 39, 42, 43], other papers merely mentioned the word “randomized” in the text. Random allocation concealment and blinding were not mentioned in all the papers. Placebo was used in one paper, but no detailed placebo information was reported [34]. No exit and lost cases were reported in the six papers that were rated as low risk of bias [8, 16, 26, 28, 34, 43]. Exit and lost cases were reported in two papers, but intention analysis was not done; therefore, it was rated as high risk of bias [6, 38]. No details were reported in other papers, and they were rated as “unclear.” Since program registrations were conducted in none of the included studies, the selection bias was “unclear” for most of the studies. However, there were discrepancies in the methodology and results in four papers, and they were rated as high risk of bias [6, 11, 16, 44]. The principle for estimating the sample amount was not reported in any of the included studies. The methodological quality of the included studies is shown in Figures 2 and 3.

### 3.4. Efficiency of Dan'e-Fukang Soft Extract in the Treatment of Endometriosis

#### 3.4.1. Efficiency

**Figure 4:**
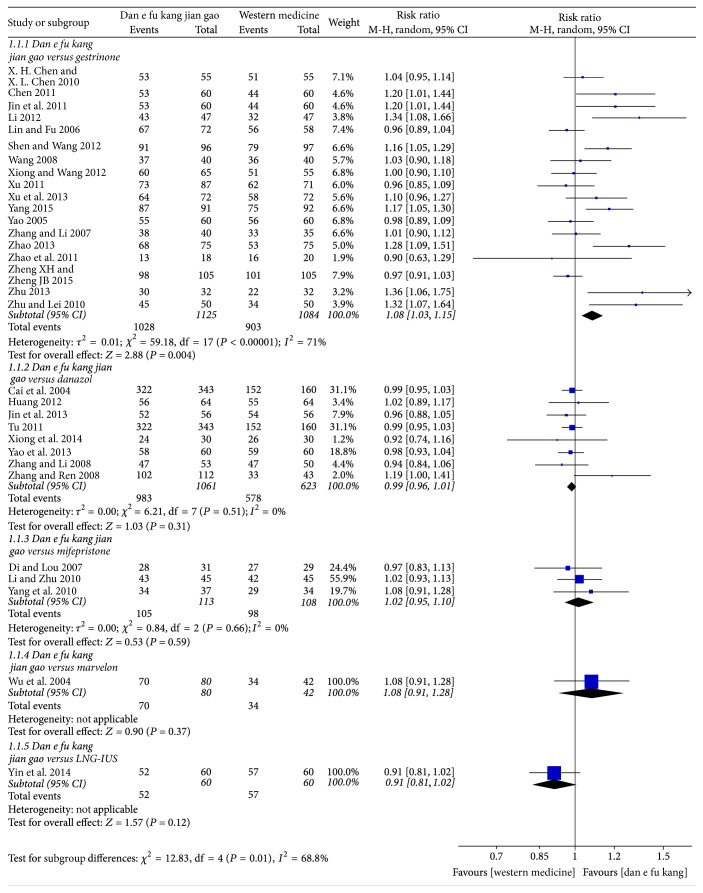
Forest plot of effective rate.

The efficiency of Dan'e-fukang soft extract and gestrinone in the treatment of endometriosis was compared in 18 papers, and there was no significant difference in the efficacy (*P* < 0.05) in 10 papers. However, meta-analysis showed that Dan'e-fukang soft extract was superior to gestrinone (RR = 1.08, 95% CI from 1.03 to 1.15, *I*^2^ = 71%, REM). The efficiency of Dan'e-fukang soft extract and danazol was compared in 8 papers, and the meta-analysis showed no significant difference in efficacy (RR = 0.99, 95% CI from 0.96 to 1.01, *I*^2^ = 0%, REM). The efficiency of Dan'e-fukang soft extract and mifepristone was compared in 3 studies, and meta-analysis showed no significant difference in efficacy (RR = 1.02, 95% CI from 0.95 to 1.10, *I*^2^ = 0%, REM). The detailed results are shown in Figure 4.

#### 3.4.2. Recurrence Rate

**Figure 5:**
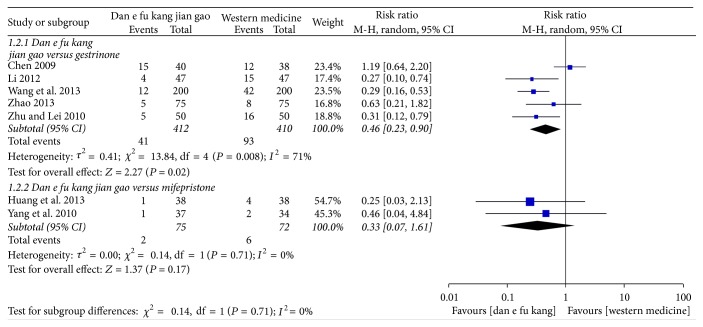
Forest plot of recurrence rate.

Meta-analysis of five papers showed that the recurrence rate of Dan'e-fukang soft extract was lower than gestrinone in the treatment of endometriosis (RR = 0.46, 95% CI from 0.23 to 0.90, *I*^2^ = 71%, REM). However, meta-analysis of two papers showed that there was no significant difference between Dan'e-fukang soft extract and mifepristone in reducing the recurrence rate of endometriosis (RR = 0.33, 95% CI from 0.07 to 1.61, *I*^2^ = 0%, REM). The detailed results are shown in Figure 5.

#### 3.4.3. Degree of Ease of Dysmenorrhea

**Figure 6:**
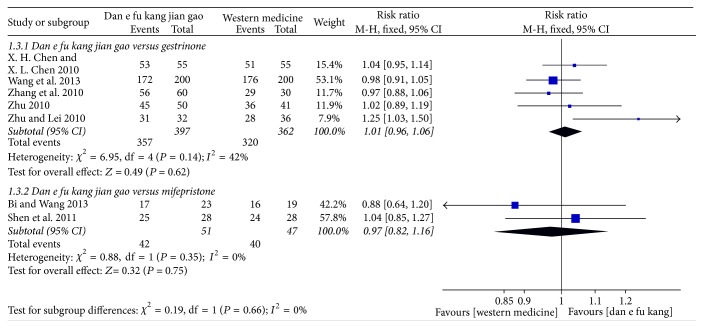
Forest plot of dysmenorrhea relieve rate.

The meta-analysis of five papers showed that there was no significant difference between Dan'e-fukang soft extract and gestrinone in the treatment of endometriosis and remission rate of dysmenorrhea (RR = 1.01, 95% CI from 0.96 to 1.06, *I*^2^ = 42%, FEM). The meta-analysis of two papers showed no difference between Dan'e-fukang soft extract and mifepristone in terms of relief of dysmenorrhea (RR = 0.97, 95% CI from 0.82 to 1.16, *I*^2^ = 0%, FEM). The detailed results are shown in Figure 6.

#### 3.4.4. CA125

**Figure 7:**
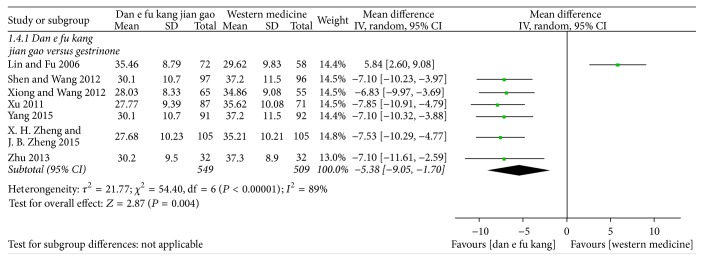
Forest plot of CA125.

Meta-analysis of seven papers showed that Dan'e-fukang soft extract was superior to gestrinone in the treatment of endometriosis by regulating CA125 (MD = −5.38, 95% CI from −9.05 to −1.70, *I*^2^ = 89%, REM). The detailed results are shown in Figure 7.

#### 3.4.5. Adverse Reactions

**Figure 8:**
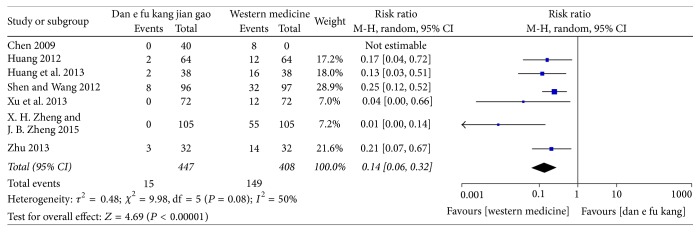
Forest plot of adverse reactions.

No significantly adverse reactions occurred in the Dan'e-fukang soft extract-treated groups in 18 papers, while adverse reactions, such as weight gain, acne, menstrual disorders, abnormal vaginal bleeding, and abnormal liver function, were reported in the western medicine-treated control groups. However, no further meta-analysis could be done because the various symptoms were not standardized. The overall incidence of adverse reactions was reported in six studies and the meta-analysis showed that the adverse reactions in the Dan'e-fukang soft extract group were less than that of the western medicine groups (RR = 0.14, 95% CI = 0.06 to 0.32, *I*^2^ = 50%, REM). The detailed results are shown in Figure 8.

## 4. Discussion

Endometriosis is a common hormone-dependent gynecological disease, mainly treated with surgeries and western medicines. The recurrence rate is high because invisible lesions cannot be effectively removed with surgeries. The commonly used drugs include gestrinone, danazol, and mifepristone. Not only are they expensive, but also have serious drug side effects, commonly causing damage to the liver and kidney. Moreover, they induce masculinity, which greatly affects the quality of life in patients.

Dan'e-fukang soft extract is composed of two key herbs: Danshen (*Salvia miltiorrhiza*) and Ezhu (*Curcuma zedoaria*).* Salvia miltiorrhiza*, well known for its characters in treating of heart and vascular diseases, has also been explored extensively for treating other diseases [45, 46], and it is documented in the United States Pharmacopeial Convention [47]. Classified by structural characteristics and chemical properties, the compounds isolated from Danshen can be categorized as water-soluble and lipid-soluble constituents [48]. Water-soluble constituents mainly exhibit cardiovascular protective activities [49–51]. The lipid-soluble constituents show properties of anticancer and anti-inflammation [52–55]. Family Zingiberaceae consisting of about 1400 species and 47 genera has been used in medicine for centuries [56]. Ezhu (*Curcuma zedoaria*) also known as white turmeric, kachur, and zedoary is a continuing herb belonging to family Zingiberaceae which is cultivated all over Asia. It is used traditionally to treat inflammation, pain, and a variety of skin ailments including wounds, as well as menstrual irregularities and ulcers [57].* Curcuma zedoaria* is being used as anti-inflammatory, carminative, antitumor, gastrointestinal stimulant, antiulcer, stomachic, antiallergic, diuretic, hepatoprotective, antinociceptive, demulcent, expectorant, rubefacient, and antimicrobial agents [57–61]. Endometriosis, based on an estrogen-inflammation dependent and blood supply disorder [62], Danshen (*Salvia miltiorrhiza*), and Ezhu (*Curcuma zedoaria*), theoretically, are better choices for the treatment on the disease.

The results of this study showed that Dan'e-fukang soft extract was superior to gestrinone in treatment of endometriosis, and its efficiency was comparable to that of danazol and mifepristone. It is important to note that adverse reactions in Dan'e-fukang soft extract group were significantly lower than the western medicine group, indicating that Dan'e-fukang soft extract can improve endometriosis to a certain degree, and it is worth further exploration. However, the quality of the literature included in this study was generally low; therefore, a firm conclusion could not be drawn.

The generally low methodological quality of the included literature is a limitation of this study. The reasons for the low quality are as follows: (1) some subjective bias may exist as no placebo-controlled and blinded trials were implemented in the studies; (2) selective reporting bias cannot be ruled out as no proposals were registered and published in the studies; and (3) no detailed randomization methods were reported in most studies and the term “randomized” was merely mentioned. Therefore, only a part of the studies is true “randomized controlled trials"; (4) the efficacy evaluation of the included studies was mainly based on compound outcomes; for example, the degree of improvement based on multiple symptoms is divided into four levels: cured, obvious effective, effective, and ineffective. Since judging criteria in the studies are inconsistent, misclassification bias might exist. In future, rigorously designed, large-scale, multicenter RCTs are recommended to further validate the efficacy of Dan'e-fukang soft extract and to draw more reliable conclusions.

## 5. Conclusion

The results of this study show that Dan'e-fukang soft extract offers certain advantages in endometriosis treatment. However, because the methodological quality of the included studies was low, rigorously designed and strictly implemented RCTs are needed to further validate its efficacy.

## Figures and Tables

**Table 1:** Characteristics of enrolling randomized controlled trials.

ID	Sample size (I/C)	Age (I/C)	Surgery	Intervention methods	Controlled methods	Treatment course	Following up	Outcomes
Bi and Wang 2013	23/19	Unreported	N/A	Dan'e-fukang, 10 g each time, 2 times a day, taken 14 days before menstruation	Mifepristone, 12.5 ng each time, 2 times a day	3 months	N/A	Dysmenorrhea, safety
Cai et al. 2004	343/160	35 (16–48)	N/A	Dan'e-fukang, 10 g each time, 2 times a day, taken 15 days before menstruation	Danazol, 200 mg each time, 3 times a day	3 months	N/A	Effective rate
Chen WZ 2010	62/58	I: 35.0 ± 3.3; C: 34.0 ± 3.1	Postoperation	Dan'e-fukang, 10 g, 1 time a day; Diphereline 3.75 mg intramuscular injection, 28 days once, 3 times totally	Diphereline, 3.75 mg intramuscular injection, 28 days once, 6 times totally	6 months	12 months	Effective rate, rate of pregnancy, recurrence rate, safety
Chen 2009	40/38	I: 30.4 ± 10.0; C: 33.4 ± 8.7	Postoperation	Dan'e-fukang, 10 g each time, 3 times a day	Gestrinone, 2.5 mg each time, 2 times a week	6 months	18 months	Rate of pregnancy, recurrence rate, safety
X. H. Chen and X. L. Chen 2010	55/55	I: 36.88 ± 5.96; C: 34.13 ± 7.25	N/A	Dan'e-fukang, 15 g each time, 2 times a day, taken 10 days before menstruation, keep taken in menstrual period	Gestrinone, 2.5 mg each time, 2 times a week	3 months	N/A	Effective rate, dysmenorrhea, safety
Chen 2011	60/60	I: 34 (21–45); C: 31 (19–46)	N/A	Dan'e-fukang, 15 g each time, 2 times a day	Gestrinone, 2.5 mg each time, 2 times a week	6 months	N/A	Effective rate, safety
Di and Lou 2007	31/29	Unreported	N/A	Dan'e-fukang, 10 g each time, 2 times a day, taken 10 days before menstruation	Mifepristone, 12.5 mg each time, 2 times a day	3 months	N/A	Effective rate, safety
Huang 2012	64/64	I: 29.15 ± 4.65; C: 28.95 ± 4.85	N/A	Dan'e-fukang, 10 g each time, 2 times a day	Danazol, 200 mg, Mifepristone, 12.5 mg each time, 3 times a day	2 months	N/A	Effective rate, CA125, safety
Huang et al. 2013	38/38	I: 37.4 ± 4.5; C: 34.5 ± 5.1	Postoperation	Dan'e-fukang, 15 g each time, 2 times a day; Mifepristone, 10 mg/d, 1 time a day	Mifepristone, 10 mg, 1 time a day	3 months	N/A	Effective rate, recurrence rate, safety
Jin et al. 2011	60/60	I: 34 (21–45); C: 31 (19–46)	N/A	Dan'e-fukang, 15 g each time, 2 times a day, taken 15 days before menstruation	Gestrinone, 2.5 mg each time, 2 times a week	6 months	N/A	Effective rate, safety
Jin et al. 2013	56/56	I: 32.6 ± 4.7; C: 31.2 ± 4.3	N/A	Dan'e-fukang, 15 g each time, 2 times a day, taken 10 days before menstruation	Danazol, 200 mg each time, 3 times a day	3 months	N/A	Effective rate, safety
Li and Zhu 2010	45/45	18–44	N/A	Dan'e-fukang, 15 g each time, 2 times a day, taken 10 days before menstruation	Mifepristone, 12.5 mg each time	6 months	N/A	Effective rate
	45/45			Dan'e-fukang, 15 g each time, 2 times a day, taken 10 days before menstruation	Marvelon, 2.5 mg, 1 time a day			
Li 2012	47/47	36.6 (22–48)	Postoperation	Dan'e-fukang, 10 g each time, 2 times a day	Gestrinone, 2.5 mg each time, 2 times a week	6 months	N/A	Effective rate, recurrence rate, safety
Lin and Fu 2006	72/58	I: 33.8 ± 7.3; C: 34.8 ± 6.1	N/A	Dan'e-fukang, 15 g each time, 2 times a day, taken 10 days before menstruation	Gestrinone, 2.5 mg each time, 2 times a week	6 months	1 year–3 years	Effective rate, CA125, rate of pregnancy, symptom score, safety
Shen et al. 2011	28/28	19–42	N/A	Dan'e-fukang, 10–15 g each time, 2 times a day; Mifepristone, 6.25 mg, 1 time a day	Mifepristone, 10 mg, 1 time a day	3 months	3 months	Dysmenorrhea, safety
Shen and Wang 2013	96/97	I: 37.0 ± 6.3; C: 36.7 ± 5.8	N/A	Dan'e-fukang, 10 g each time, 2 times a day	Gestrinone, 2.5 mg each time, 2 times a week	6 months	N/A	Effective rate, CA125, safety
Tu 2011	343/160	35 (16–48)	N/A	Dan'e-fukang, 10 g each time, 2 times a day, taken 15 days before menstruation	Danazol, 200 mg, 3 times a day	3 months	N/A	Effective rate, safety
>Wang et al. 2013	200/200	Unreported	N/A	Dan'e-fukang, 10–15 g each time, 2 times a day	Gestrinone, 2.5 mg each time, 2 times a week	3 months	1 year	Dysmenorrhea, recurrence rate, safety
Wang 2008	40/40	I: 28.15 (19–45); C: 30 (21–43)	N/A	Dan'e-fukang, 10 g each time, 2 times a day,	Gestrinone, 2.5 mg each time, 2 times a week	3 months	N/A	Effective rate, safety
Wang and Hu 2007	30/30	I: 30 ± 3.5; C: 29 ± 4.4	Postoperation	Dan'e-fukang, 15 g each time, 2 times a day	No treatment	2 months	12 months	Effective rate, rate of pregnancy, recurrence rate, safety
Wu et al. 2004	80/42	I: 32 (23–41); C: 32.5 (24–40)	N/A	Dan'e-fukang, 10–15 g each time, 2 times a day	Marvelon, taken on the fifth day of menstruation, 1 tablet a day	3 months	N/A	Effective rate, improvement of symptoms
Xiong et al. 2014	30/30	I: 27.3 ± 1.9; C: 29.1 ± 2.7	Postoperation	Dan'e-fukang, 10 g each time, 2 times a day	Danazol, 200 g each time, 3 times a day	6 months	6 months	Effective rate, recurrence rate, safety
Xiong and Wang 2012	65/55	I: 34.3, C: 33.8	N/A	Dan'e-fukang, 15 g each time, taken 10 days before menstruation, 2 times a day	Gestrinone, 2.5 mg each time, 2 times a week	3 months	3 months	Effective rate, CA125, rate of pregnancy, safety
Xu 2011	87/71	I: 36.21 ± 5.25; C: 36.96 ± 5.31	N/A	Dan'e-fukang, 15 g each time, taken 10 days before menstruation, 2 times a day	Gestrinone, 2.5 mg each time, 2 times a week	3 months	3 months	Effective rate, dysmenorrhea, CA125, rate of pregnancy, safety
Xu et al. 2013	72/72	25–49	N/A	Dan'e-fukang, 15 g each time, 2 times a day	Gestrinone, 2.5 mg each time, 2 times a week	3 months	N/A	Effective rate, dysmenorrhea, safety
Yang 2015	91/92	I: 37.01 ± 6.3; C: 36.7 ± 5.8	N/A	Dan'e-fukang, 10 g each time	Gestrinone, 2.5 mg each time, 2 times a week	6 months	N/A	Effective rate, CA125, rate of pregnancy, safety
Yang et al. 2010	37/34	Unreported	Postoperation	Dan'e-fukang, 10 g each time, 2 times a day	Mifepristone, 10 mg each time, 1 time a day	6 months	N/A	Effective rate, rate of pregnancy, recurrence rate, safety
Yao 2005	60/60	I: 28.5 (23–40); C: 30 (21–43)	N/A	Dan'e-fukang, 10 g each time, 2 times a day, taken 10 days before menstruation	Gestrinone, 2.5 mg each time	6 months	N/A	Effective rate, safety
Yao et al. 2013	60/60	36 (18–50)	N/A	Dan'e-fukang, 15 g each time, 2 times a day, taken 15 days before menstruation	Danazol, 200 mg each time, 3 times a day	3 months	N/A	Effective rate
Yin et al. 2014	60/60	I: 34.9 ± 7.1; C: 35.9 ± 5.9	N/A	Dan'e-fukang, 10 g each time, 2 times a day	Intrauterine placed Mirena	9 months	N/A	Effective rate
Zhang et al. 2010	60/30	33.4 (20–53)	N/A	Dan'e-fukang, 10–15 g each time, 2 times a day, taken 10 days before menstruation	Gestrinone, 2.5 mg each time, 2 times a week;	3 months	N/A	Dysmenorrhea, safety
	60/20			Dan'e-fukang, 10–15 g each time, 2 times a day, taken 10 days before menstruation	Placebo			
Zhang and Li 2008	53/50	36 (18–50)	N/A	Dan'e-fukang, 10 g each time, 2 times a day	Danazol, 200 mg each time, 3 times a day	3 months	N/A	Effective rate, safety
Zhang and Li 2007	40/35	I: 33.0 ± 2.7; C: 34.0 ± 5.4	Postoperation	Dan'e-fukang, 10 g each time, 2 times a day	Gestrinone, 2.5 mg each time, 2 times a week	3 months	N/A	Effective rate, rate of pregnancy, safety
Zhang and Ren 2008	112/43	34.6 (19–48)	N/A	Dan'e-fukang, 15 g each time, 2 times a day,	Danazol, 200 mg each time, 2 times a day	3 months	3 months	Effective rate
Zhao 2013	75/75	I: 35.9 ± 5.68; C: 36.7 ± 6.8	Postoperation	Dan'e-fukang, 10 g each time, 2 times a day	Gestrinone, 2.5 mg each time, 2 times a week	7 months	N/A	Effective rate, recurrence rate, safety
Zhao et al. 2011	18/20	21–42	Postoperation	Dan'e-fukang, 15 g each time, 2 times a day, taken 10 days before menstruation	Gestrinone, 2.5 mg each time, 2 times a week	6 months	1 year	Effective rate, hormone level
X. H. Zheng and J. B. Zheng 2015	105/105	I: 36.25 ± 4.35; C: 36.05 ± 4.01	N/A	Dan'e-fukang, 15 g each time, 2 times a day	Gestrinone, 2.5 mg/d, the first taken on the first day of menstruation, the second taken on the fourth day of menstruation	3 months	N/A	Effective rate, dysmenorrhea, CA125, rate of pregnancy, safety
Zhu 2010	50/41	I: 36.19 ± 4.66; C: 37.34 ± 4.98	N/A	Dan'e-fukang, 15 g each time, 2 times a day	Gestrinone, 2.5 mg each time, 2 times a week	3 months	3 months	Dysmenorrhea, safety
Zhu 2013	32/32	35.8 ± 2.3	N/A	Dan'e-fukang, 10 g each time, 2 times a day	Gestrinone, 2.5 mg each time, 2 times a week	6 months	N/A	Effective rate, CA125, safety
Zhu and Lei 2010	50/50	36.19 ± 4.84	Postoperation	Dan'e-fukang, 10 g each time, 2 times a day, taken 1 week after operation	Gestrinone, 2.5 mg each time, 2 times a week, taken 1 week after operation	6 months	3 years	Effective rate, dysmenorrhea, recurrence rate, improvement of symptoms
